# Solution structure and biophysical characterization of the multifaceted signalling effector protein growth arrest specific-1

**DOI:** 10.1186/s12858-015-0037-6

**Published:** 2015-02-28

**Authors:** Katja Rosti, Adrian Goldman, Tommi Kajander

**Affiliations:** Institute of Biotechnology, Structural Biology and Biophysics, University of Helsinki, Helsinki, Finland; Astbury Centre for Structural Molecular Biology, School of Biomedical Sciences, University of Leeds, Leeds, UK; Department of Biosciences, Division of Biochemistry, University of Helsinki, Helsinki, Finland

**Keywords:** GAS1, Growth arrest specific-1, Solution X-ray scattering, Protein structure, RET, Sonic hedgehog

## Abstract

**Background:**

The protein growth arrest specific-1 (GAS1) was discovered based on its ability to stop the cell cycle. During development it is involved in embryonic patterning, inhibits cell proliferation and mediates cell death, and has therefore been considered as a tumor suppressor. GAS1 is known to signal through two different cell membrane receptors: Rearranged during transformation (RET), and the sonic hedgehog receptor Patched-1. Sonic Hedgehog signalling is important in stem cell renewal and RET mediated signalling in neuronal survival. Disorders in both sonic hedgehog and RET signalling are connected to cancer progression. The neuroprotective effect of RET is controlled by glial cell-derived neurotrophic factor family ligands and glial cell-derived neurotrophic factor receptor alphas (GFRαs). Human Growth arrest specific-1 is a distant homolog of the GFRαs.

**Results:**

We have produced and purified recombinant human GAS1 protein, and confirmed that GAS1 is a monomer in solution by static light scattering and small angle X-ray scattering analysis. The low resolution solution structure reveals that GAS1 is more elongated and flexible than the GFRαs, and the homology modelling of the individual domains show that they differ from GFRαs by lacking the amino acids for neurotrophic factor binding. In addition, GAS1 has an extended loop in the N-terminal domain that is conserved in vertebrates after the divergence of fishes and amphibians.

**Conclusions:**

We conclude that GAS1 most likely differs from GFRαs functionally, based on comparative structural analysis, while it is able to bind the extracellular part of RET in a neurotrophic factor independent manner, although with low affinity in solution. Our structural characterization indicates that GAS1 differs from GFRα’s significantly also in its conformation, which probably reflects the functional differences between GAS1 and the GFRαs.

## Background

Growth Arrest Specific-1 gene (GAS1) was found in a screen to identify genes specifically expressed in growth-arrested mouse cells [1]. The full-length cDNA of human GAS1 was cloned [2,3] and the mature protein was found to contain 345 amino acids, a potential signal peptide, one N-glycosylation site at Asn117 and an aminated Ser318 [2,3]. The aminated Ser318 allows the mature protein to be glycophosphatidylinositol (GPI) anchored to the cell membrane [2,4].

GAS1 was found to arrest cell cycle by stopping the cells in synthesis (S) phase [1,5] and due to its ability to arrest cell proliferation in p53-dependent manner it has been considered to be a tumour suppressor protein [6,7]. Generally GAS1 might act as a tumour suppressor in adult brain, though the expression in brain leading to apoptosis has not been observed in adults [3,8]. Sequence comparison of human and murine GAS1 genes suggested that it has a conserved RGD-peptide sequence for possible RGD-dependent integrin binding at residues 306–308 [3].

Additionally GAS1 has been shown to have a significant role in development [9]. At early developmental stages GAS1 is expressed in most embryonic tissues. During development GAS1 has been reported to inhibit cell proliferation and to mediate cell death, to be involved in embryonic patterning, and to support growth of the cerebellum [3,8].

GAS1 is clearly a multifunctional protein, since it signals through at least two different kinds of transmembrane receptor proteins, Rearranged during transformation (RET) [8] and the Hedgehog receptor protein patched-1 [10,11]. The Hedgehog signalling pathway is important in development, stem cell renewal, and cancer progression. GAS1 is able to bind sonic hedgehog (SHH) and activate the signalling pathway from patched-1 [10,11]. RET, on the other hand, is a transmembrane kinase, first identified as a proto-oncogene [12]. Overactivity of RET can cause several types of cancers, and loss-of-function mutations cause varying degrees of loss in the enteric nervous system resulting in Hirschprung’s disease (see *e.g.* Robertson and Mason [13]). Normally RET mediated signalling is controlled by Glial cell-derived neurotrophic factor family ligands (GFLs) and Glial cell-derived neurotrophic factor receptor alphas (GFRαs), which form a four-member protein family (GFRα1-4) [14].

Of these, GAS1 has highest (28%) similarity to GFRα1, while GAS1 and GFRα4 both have only two domains unlike GFRα1-3, which consists of three domains [15]. The secondary structure of mammalian GAS1 is predicted to be mostly α-helical separated by short β-strands and to have a long unstructured C-terminal domain [15]. By binding GFLs, GFRαs take part in controlling the survival of neurons, neuron branching, and functional recovery [14]. The most studied member of GFLs is GDNF, which was identified due to its function as a survival factor for midbrain dopaminergic neurons [14]. GDNF forms a complex with GFRα1 and promotes the survival of neurons [16]. GFLs, in general, are dimeric proteins and they are capable of binding two GFRα receptors per ligand [14]. After the formation of GFRα-GFL complex, the complex then binds to the transmembrane tyrosine kinase RET [16].

Despite the structural similarity to GFRαs, GAS1 differs from them functionally because it is able to bind to RET in a ligand independent way [8]. In addition, the intracellular signalling pathway is most probably different than for GFRα-GDNF complex, and GAS1 bound to RET blocks AKT activation, and increases ERK activation [8].

GAS1 has been suspected to be an ancestor of GFRα proteins [8,15,17]. Thus the four GFLs and GFRαs could have been generated by genome duplications at the origin of vertebrates, and at this point the gene encoding GAS1 could have diverged from GFRα-like proteins [17,18].

It has been hypothesised that the relative abundance and localization of GFRαs, GFLs and GAS1 could determine in certain conditions whether cells survive or die [15]. Furthermore, GAS1 expression is increased in neuronal cell death during early development [19]. Therefore, GAS1 could work as a switch between proliferation and differentiation in neuronal development [8]. GAS1 has been shown to colocalize to lipid rafts with RET [8]. This has led to the hypothesis that GAS1 could be a negative modulator of GDNF signalling and able to control GDNF stimulation *via* RET [8,20].

## Results

### Production and purification of human GAS1 protein

**Figure 1:**
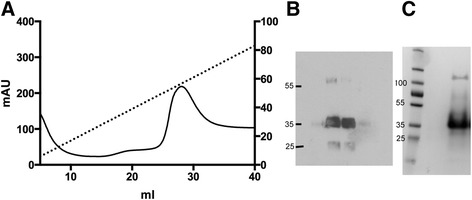
**Purification of recombinant GAS1 from insect cells. A)** Ni-affinity chromatogram for GAS1 purification **B)** Western blot of fractions from the Ni-affinity chromatography peak at *ca.* 28 ml. Fractions of 1 ml were collected, and fractions from peak area at 23, 27, 29 and 33 ml were tested in the western blot. **C)** SDS-PAGE analysis of GAS1 purification (left lane, molecular weight marker, with sizes indicated; right lane, purified GAS1 after gel filtration; the gel was Coomassie stained.).

After cloning and expressing human GAS1 in *Tricoplusia Ni* cells, we purified secreted GAS1 from the insect cell growth medium using Ni-affinity chromatography (Figure 1), and the identity and size of the expressed protein was verified with a western blot (Figure 1). The purified protein is glycosylated and therefore does not run exactly according to excepted molecular weight on the SDS-PAGE, but slightly higher. Thrombin was used to cleave off the tags, and the size of the protein after cleavage was verified by SDS-PAGE and MALDI-TOF mass spectroscopy. The yield of purified protein was on average ca. 1 mg/L.

**Figure 2:**
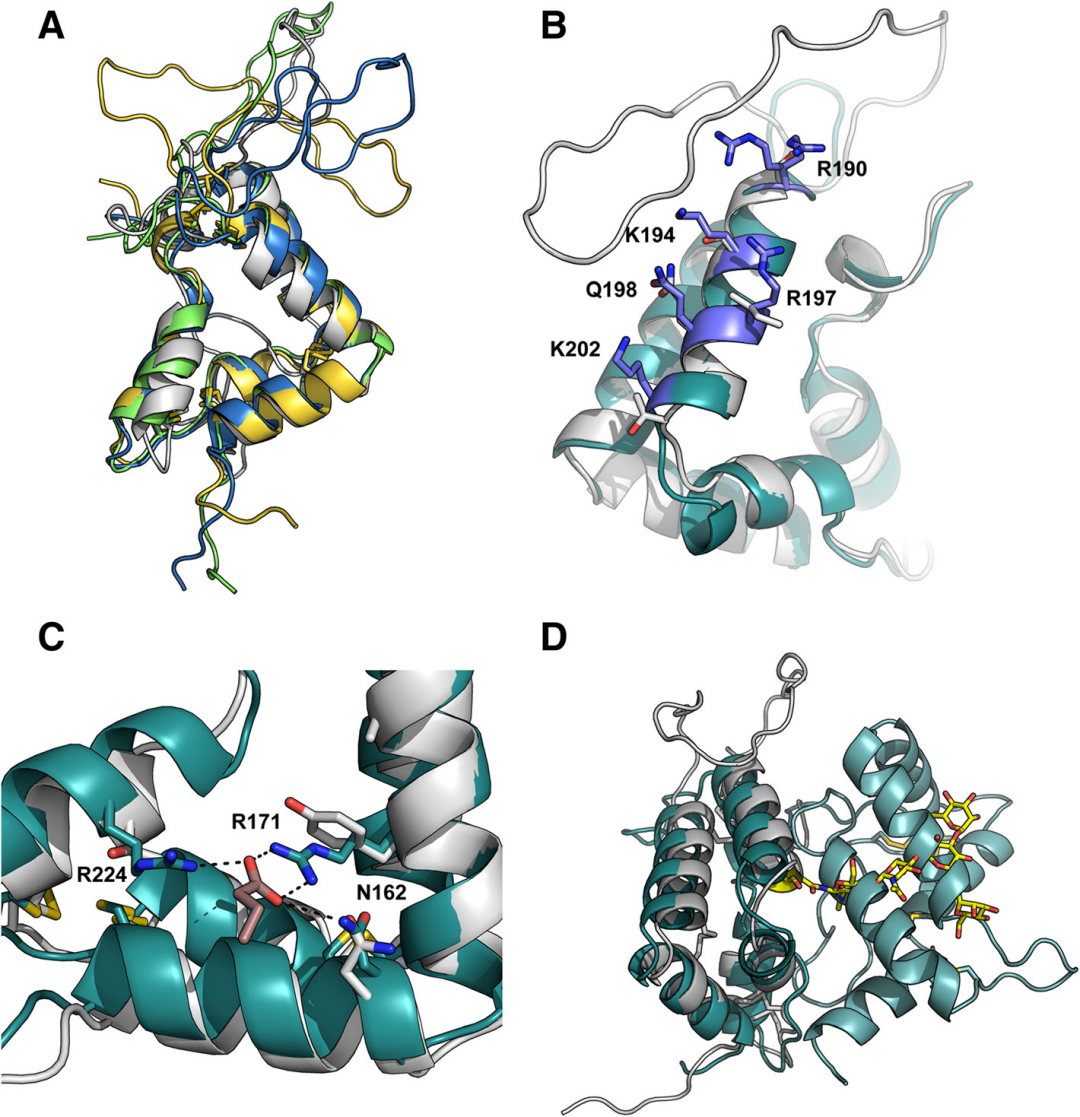
**Homology modelling of human GAS1 and comparison to GDNF receptor structures. A)** Four representative homology models of GAS1 N-terminal domain showing different orientations and flexibility of the extended intradomain loop of higher vertebrate proteins. The models were generated with the Raptor-X server, as mentioned in the text. The template for modelling was the GFRα1 structure (PDB: 2VE5). **B)** comparison to N-terminal domain of GFRα1 (dark cyan) and N-terminal domain (GAS1, grey) and RET/heparin binding site (grey residues, GAS, cyan residues, residues involved in heparin and indicated as putative RET binding residues). GFRα1 RET/heparin binding-site residues are labelled. **C)** The GDNF binding site residues GFRα1 (dark cyan) vs. GAS1 (grey)**.** The GDNF Glu binding to the GFRα1 is colored brown, and hydrogen bonds are indicated with dashed lines. A Tyr and Ser residue occupy the positions in GAS1 equivalent to GFRα1 Arg171 and 224, GFRα1 GDNF binding residues are labelled. Disulphides in the figure are shown with stick presentation and atoms S atoms in yellow **D)** Position of the N-glycosylation site in GAS1 vs. GFRα1 domain interface of domains D2 and D3. N-glycan at Asn117 of GAS1 is indicated in yellow with a stick presentation, the GFRα1 D2 and D3 domains are depicted in dark cyan, GAS1 N-terminal domain is drawn in grey.

Based on the primary sequence one N-glycosylation site was predicted at Asn117 located in the N-terminal domain. The corresponding site in the GFRα-structures is located at the domain interface between the two homologous disulphide rich domains, in a tightly packed two-domain structure [21,22], suggesting that the GAS1 overall conformation is quite likely very different (see below, Figure 2).

When the protein was treated with PNGase F to remove glycans, the size of the protein diminished slightly on SDS-PAGE (data not shown), and based on MALDI-TOF analysis, we observe a decrease in molecular weight of *ca.* 900 Da; the glycosylated protein had a molecular mass of 29.8 kDa and the de-glycosylated protein of 28.9 kDa, according to MALDI-TOF, while the theoretical molecular mass of the protein without glycosylation is 29.0 kD, thus matching well with the mass spectrometry results. The result obtained for glycosylated protein corresponds to approximately one N-glycan added post-translationally in the insect cells. The purified protein was functional in binding to RET *in vitro*, and found to be over 90% pure on SDS-PAGE, and monodisperse in solution after gel filtration.

### GAS1 is a monomer is solution and highly thermostable

**Figure 3:**
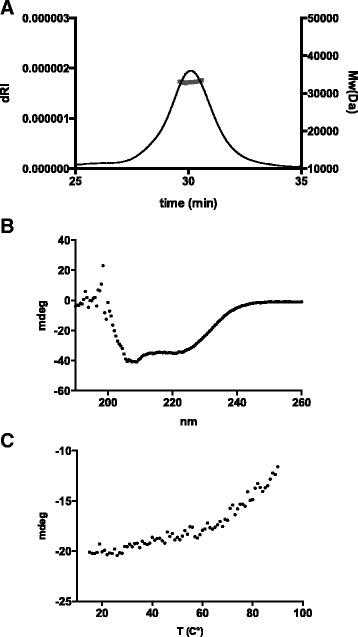
**GAS1 analytical gel filtration, circular dichroism and thermal unfolding. A)** GAS1 sample was run on Superdex 200 10/300 gel filtration column in TBS at 0.5 ml/min, at protein concentration of 1 mg/ml. A single major peak at 30 min (X-axis) eluted and based on multi-angle light scattering had molecular weight (right Y-axis) of *ca.* 33 kDa, matching relatively well with theoretical molecular weight of the monomer. The peak is plotted as a function of dRI signal (left Y-axis). **B)** The CD spectrum of GAS1 and **C)** the residual thermal denaturation of GAS1 as monitored by CD at 222 nm.

The cleaved, non-tagged protein was found to be a monomer by analytical size exclusion chromatography and multi-angle light scattering (SEC-MALLS) (Figure 3). At 1 mg/ml and 4.5 mg/ml the SEC-MALLS runs gave a single peak with molecular mass of *ca.* 31–33 kDa (Figure 3), matching quite well to the theoretical size of monomeric GAS1 (29.0 kDa) considering the additional glycosylation at one site. Similarly, based on the small-angle X-ray scattering (SAXS) data, the molecular weight matches most closely to a monomer (Table 1). In our opinion, this is likely to reflect the oligomerization state of the lipid-anchored protein, which is unlikely to be affected by the anchor. No detectable oligomerization was observed in native PAGE or gel filtration at 4.5 mg/ml, while in SAXS data an effect from residual aggregation was evident at higher concentrations.

**Table 1:** **SAXS-derived size parameters for GAS1**

I(0) (Guinier)	25.08
I(0) (Porod)	24.8
D_max_ (nm)	10.5
R_g_ (Guinier/nm)	3.01
R_g_ (Porod/nm)	3.00
Porod volume (V_p_)	54.2
M_w_(theoretical)	29 158.3 g/mol
M_w_(calc) (Guinier)	25.1 kDa
M_w_(calc) (Porod vol.)	31.9 kDa (V_p_/1.7)

The Guinier I(0)-value was calculated against an absolute reference (scattering of water relative to sample) [23] and the I(0) for the sample is then equal to the molecular weight. Molecular weight from the Porod volume is estimated according to Petoukhov et al. [24].

Circular dichroism (CD) spectroscopy was used to verify the secondary structure content of GAS1 and, as expected, the CD spectrum was typical for an α-helical protein (Figure 3). A measured temperature denaturation curve with CD gave a result with partial melting of the structure when heated to 90°C (Figure 3). However, full temperature denaturation was not possible to obtain by CD, nor by differential scanning calorimetry (data not shown), possibly due to the high disulphide content of the protein. This suggests that the domain structure is thermally very stable. The decrease in CD signal at 222 nm did not even reach the midpoint of denaturation when heated to 90°C (Figure 3).

### Sequence analysis and evolution of GAS1

**Figure 4:**
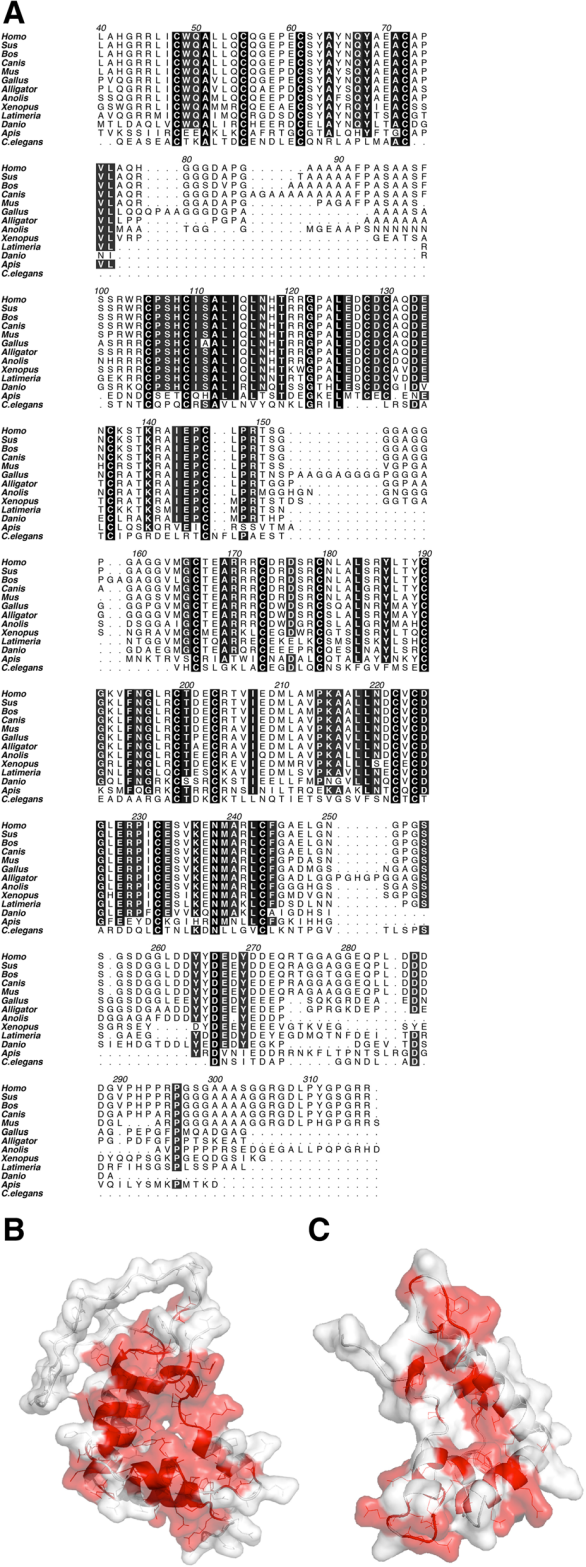
**The GAS1 family sequence alignment. A)** The sequences start from the beginning of the mature human GAS1 and numbered according to the human amino acid numbering. Residues over *ca.* 85% conserved (11/13) are coloured with a black-to-grey scale, in higher vertebrates (mammals) there is an extended loop in the N-terminal domain around residues 80–100 (human GAS1 numbering). The C-termini are poorly conserved (residues beyond 250), note the RGD sequence at 306–308. **B)** Conserved surface features on GAS1 displayed on the N-terminal domain. **C)** Conserved surface features on GAS1 displayed on the C-terminal domain; conserved sites in B and C are coloured in red (with >75% sequence similarity, see text).

The GAS1 protein domain structure is defined by two GFRα–like domains, with 10 conserved disulphide bridge-forming cysteines in each domain [25]. GAS1 is present in all the vertebrates, and homologs are also found in lower chordates (*e.g. Ciona* and *Amphioxus* [17]). In addition, GAS1 homologs also occur in *C. elegans* and honeybee, but not, surprisingly, in *Drosophila*. The sequence identity to vertebrate proteins, however, is quite low: *ca.* 21-24% for honeybee and only 14-19% for the worm sequence (Table 2). Two conserved cysteines are missing from the *C. elegans* sequence (Figure 4), and thus the protein fold might not be fully conserved in the *C. elegans* homolog (phas-1) [26]. Alignment of the GAS1 sequences shows that, in higher vertebrates, the N-terminal domain has an insertion with low sequence complexity (Figure 4), apparently forming an extended loop structure (Figure 2). Mammals have also an RGD sequence in the C-terminal linker region. Also, a single N-glycosylation site at Asn117 is predicted to be conserved based on sequence in all vertebrates, while it is not present in the invertebrates. In the set of conserved residues beyond the structural cysteines (or positions with highly conserved mutations *e.g*. Asp to Glu) (Figure 4) a subset are present only in the vertebrate proteins, and, other than the conserved cysteines, only ten residues are conserved also in *C. elegans*.

**Table 2:** **Amino acid sequence identities (%) within the GAS1 protein family**

	**Human**	**Sus scrofa**	**Bos Taurus**	**Canis Lupus**	**Mouse**	**Gallus gallus**	**Alligator**	**Anolis**	**Xenopus**	**Latimeria**	**Danio rerio**	**Apis**	**C. elegans**
**Human**	100												
**Sus scrofa**	**95.3**	100											
**Bos Taurus**	**94.1**	**94.4**	100										
**Canis Lupus**	**91.1**	**91.4**	**90.2**	100									
**Mouse**	**85.4**	**85.4**	**84.9**	**82.9**	100								
**Gallus gallus**	61.9	61.7	60.1	62.1	59.6	100							
**Alligator**	60.3	60.7	59.9	59.9	60.1	**70.9**	100						
**Anolis**	55.8	56.2	54.5	57	55.7	54.7	57.6	100					
**Xenopus**	51.2	51.7	50.8	50.6	50.5	52.2	55	48.4	100				
**Latimeria**	47.2	47.4	46.6	46.3	45.9	48.4	50.5	42.1	49	100			
**Danio rerio**	35.2	34.5	35	35.6	35.8	37	38.7	34.2	37.6	43.4	100		
**Apis**	22.1	21.7	21.3	21.6	21.1	23.1	23.7	22.6	22.1	22.8	23.7	100	
**C.elegans**	15.6	15.6	14.6	15.5	14.3	14.7	15.9	14.2	19	17.8	17.1	15.4	100

Pairwise identities between species that are over 70% are shown in bold.

When the conserved amino acid residues are displayed onto the surface of the modelled domains, the most conserved surface patch is found on the N-terminal domain surface formed by residues on helices 3–5, whereas the C-terminal domain surface did not reveal large patches of conservation (Figure 4). Here conservation is defined by >75% sequence similarity amongst the residue groups KHR, ED, NQSTGP, ILMVCA and FYW.

### Homology modelling of GAS1 domains and comparison to GFRαs

We constructed homology models of both GAS1 domains with the RaptorX-server (http://raptorx.uchicago.edu/) [27], designed for low sequence homology-based modelling. The models for both domains fit well to the GFRα-structure (PDB: 2VE5) [22]. As described above, the N-terminal domain of mammalian GAS1-proteins contain a large inserted loop with low sequence complexity, which based on modelling indeed appears to form a large flexible loop, but whether this region has functional significance or not remains unclear.

Modelling of GAS1 has partially been done before also by Cabrera *et al*. [8] and Schueler-Furman *et al.* [15]. Here our aim was to study possible conservation of the ligand binding regions of GFRαs vs. GAS1, to do more detailed analysis on the structure, and to provide models for the analysis of SAXS data (see below).

Although the sequence identity to the related GFRα structures is low, the cysteines involved in disulphide bridges are well conserved for the two GFRα-type domains and make structure prediction possible. The N-terminal domain of GAS1 is equivalent of the second domain in GFRαs, which contains the growth factor binding site. We aligned our model of GAS1 with the GFRα1:GDNF complex structure [22], and, based on the structural alignment, the binding region for the GDNF is not conserved in GAS1 (Figure 2). Similarly the conserved binding residues in GFRα2:Artemin complex (PDB: 2GH0) [21], are not present in GAS1. In fact, the key ionic residues required for ligand binding are conserved in both these structure, but not present in GAS1. The conserved key residues for GDNF binding in GFRα1 are Arg171, Arg224 and Asn162. In our structural analysis GAS1 has Tyr26, Thr100, Gln17 in equivalent side chain positions; in GAS1, the ion triplet required for growth factor binding [22] is absent.

**Figure 5:**
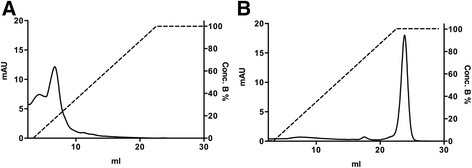
**Heparin affinity chromatography of GAS1. A)** Elution of GAS1 as a function of salt concentration. **B)** Elution of HBGAM as a function of salt concentration. Chromatograms are plotted with absorbance in mAU unit (right y-axis) and salt concentration gradient to 1 M NaCl (%) (left y-axis), against volume in ml.

It has also been suggested by Wang et al. [21] and Parkash et al. [22] that a RET binding region would be located mostly in the second (“D2”) domain on the GFRαs and would involve the GFRα1 residues Arg190, Lys194, Arg197, Gln198, Lys202, Arg257, Arg259, Glu323, and Asp324. This site forms a highly positively charged patch on the surface of GFRα1, identified also as a heparin binding site by Parkash et al. [22]. We analysed the equivalent region in the N-terminal domain of GAS1, but found no conservation between the GFRα1 structure and GAS1 (Figure 2). Overall GAS1 is not positively charged, as would be expected of a typical heparin-binding molecule. The calculated pI-value for human GAS1 is 5.0 whereas for human GFRαs the values range from 7.5-7.6 (GFRα2 − 3) to 8.4 (GFRα1) and 10.1 (GFRα4). Also, GAS1 does not contain a highly positively charged patch in the suggested RET/heparin binding region, and heparin affinity chromatography of GAS1 showed no significant binding to the column (Figure 5), whereas the well-known heparin binding protein HBGAM eluted only at 1 M NaCl (Figure 5). Finally, modelling of GAS1 N- terminal domain shows that glycosylated Asn117 will be situated at the position equivalent to the domain interface between domains D2 and D3 in the GFRα structures (Figure 2).

### Structural characterization of GAS-1 by solution X-ray scattering

**Figure 6:**
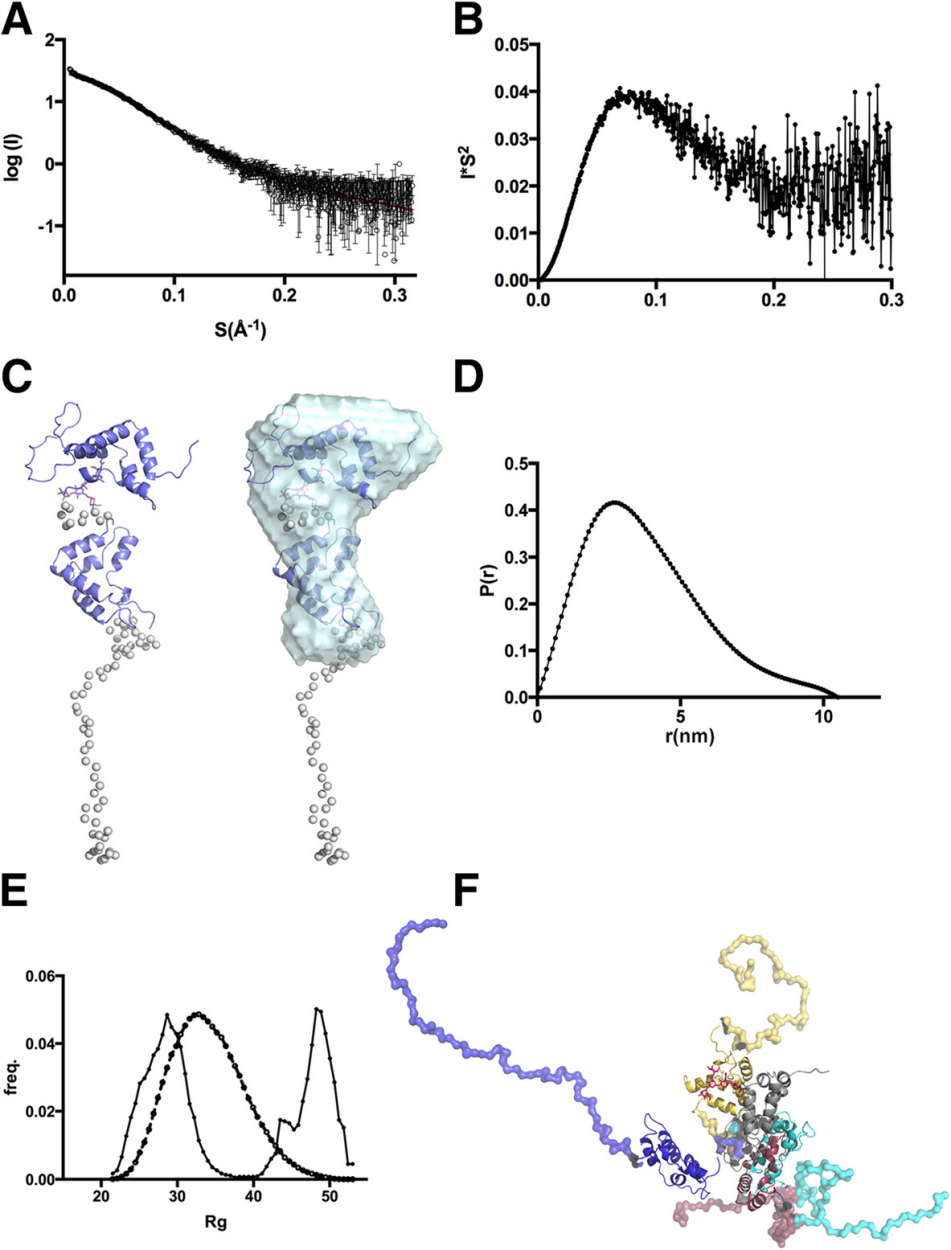
**Rigid body and ab initio modelling of GAS1 based on SAXS data. A)** Scattering curve and fit of CORAL rigid body model (red line) to observed data. **B)** The Kratky-plot from the experimental data, suggesting a folded structure with some flexibility. **C)** CORAL generated model with N- and C-terminal domains as rigid bodies (blue) with flexible linker regions (grey beads; left), and the ab initio model for GAS1 generated by DAMMIN (green; average of 10 calculations) fitted over the rigid body model (right). **D)** The distance distribution calculated for GAS1 SAXS data. **E)** SAXS ensemble modelling of GAS1 solution conformations shown as the statistical distribution of R_g_-values of best fitted models (continuous line with closed circles) vs. initial random pool (dashed line with open circles) shows a bimodal distribution of GAS1 solution conformations. **F)** The selected pdb-files representing the ensemble with Chi^2^ = 0.84 fit to the experimental data, showing extended (blue) and more collapsed models (yellow, red, cyan) in the final ensemble; the N-terminal domain (in grey) was fixed relative to the rest of the protein during the runs. The modelled glycan structure is shown as red “stick” presentation on the N-terminal domain.

Solution X-ray scattering (SAXS) data indicated that GAS1 is monomeric at 0.8 mg/ml in solution based on the Porod volume and Guinier plots (Table 1): at higher concentrations the protein starts to aggregate, and the data beyond 1 mg/ml could not be analysed. Rigid body modelling of the structure was done based on homology models of the individual domains, and elongated models gave the best fits (Figure 6). We also calculated *ab initio* envelopes, which matched well with rigid body modelling of the structure (Figure 6). Both differ significantly from the compact GDNF co-receptor structures [21,22]. However, as it is clear that the structure is likely to be flexible, in particular the C-terminal long unstructured region, we also did ensemble fitting of the model against the data. This resulted in a bimodal ensemble represented by four major structures selected from the initial random pool of 10 000 structures, which fit to the data with χ^2^ = 0.84 (Figure 6). The selected structures represent states with extended and collapsed C-terminal linkers and variable orientations of the domains relative to each other (Figure 6). Taken together it appears from the SAXS data that the orientation of the domains of GAS1 relative to each other is not fixed; clearly the protein exists in two populations of extended and collapsed conformations.

### Binding and affinity of GAS1 to RET *in vitro*

**Figure 7:**
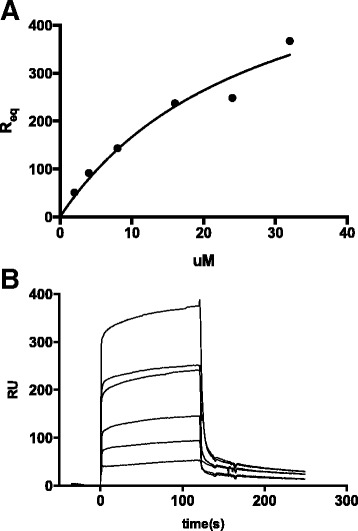
**Binding of human GAS1 to RET. A)** A binding curve of GAS1 to ecRET. Purified human GAS1 shows clear ligand independent binding to ecRET. Binding was measured with a concentration series of 2 μM, 4 μM, 8 μM and 16 μM, 24 μM and 32 μM. The dissociation constant (*K*_d_) was obtained based on the equilibrium (*R*_eq_) values at different concentration from two independent experiments with a *K*_*d*_ = 12.2 ± 8.2. **B)** The biacore sensograms for GAS1 binding (in response units, RU) to RET at different concentrations (as above).

We tested whether GAS1 is able to directly interact with RET in a ligand independent way, as previously reported [8]. For this purpose, and to determine the affinity of the interaction, the RET receptor was coupled to a chip for surface plasmon resonance assay, and binding of a concentration series of GAS1 to immobilized RET was measured. A *K*_d_-value of 12.2 ± 8.2 μM was measured for the interaction *in vitro* (Figure 7). The kinetics of the interaction were too fast to allow for measurement of on- and off-rates, as is evident from the time scale of the binding and dissociation from the sensograms (Figure 7).

## Discussion

We have overexpressed and purified the human GAS1 protein in soluble form without the GPI-anchor, and biophysically characterized the protein. We constructed homology models for both domains of GAS1 and were able to analyse the domain structure of the protein in comparison to the structurally related GFRαs. As reported earlier by others [8], GAS1 has clearly two GFRα-like domains, but we have shown here that GAS1 differs significantly from GFRαs in both sequence and in structure.

The differences can be characterized as follows: Firstly, GAS1 has a large, 10–15 amino acid unstructured, low complexity Ala/Gly/Pro-containing loop in the N-terminal domain. This loop region is present in higher vertebrates, in mammalians and chicken, but not in fish (Figure 4). Whether this loop might have some function remains unknown. Secondly, the two-domain structure of GAS1 appears to be more flexible overall than in the characterized GFRα structures. In the GFRαs, the functional domains D2 and D3 form a compact structure, whereas GAS1 SAXS analysis reveals a flexible ensemble of structures, with the N- and C-terminal domains as independent structural units. This might reflect the location of the functional binding regions of the molecule *versus* those of the GFRαs. As expected, the protein is α-helical based on the CD spectrum. It is somewhat intriguing that we were not able to fully denature the protein; apparently the disulphide-linked arrangement of the domains is highly thermostable, and this might be a general feature of the GFRα-family.

The structural flexibility is probably a conserved feature in the protein family, as the single N-glycosylation site in the human protein is conserved in chordates. This N-glycan blocks the GFRα-equivalent domain interface, and hence the formation of that type of compact structure. It has been also observed that this glycosylation site might have functional significance for SHH binding [28].

Our sequence analysis and that by Hätinen *et al*. [17] suggest that GAS1 is conserved during evolution, with homologs in chordates (from *Ciona* and *Amphioxus*), arthropods and roundworms, thus possibly representing an ancestral GFRα-like protein [17] However, the sequence identity from chordate to invertebrates (*e.g.* honey bee and *C. elegans*) genes is low, 14-19% for the worm phas-1 homolog of GAS1 [26], and it remains an open question whether the insect or worm genes identified as GAS1 actually share any of the functions of vertebrate GAS1/GFRα type of receptors, either in RET or the hedgehog signalling.

GAS1, as well as GFRα-like proteins, are conserved beyond vertebrates, while GFLs are not expressed in non-vertebrates. This suggests that either RET binding, independent of GFLs is conserved, or that there are alternative receptors for GAS1. In case of GAS1, this could be SHH and patched-1. Interestingly in *Drosophila*, the GRFα-like protein does not interact with RET but does interact with Drosophila NCAM analog, FasII [29]. The mammalian GFLs are known to be ligands of NCAM [30]: whether GAS1 might interact with NCAM homologs remains to be investigated.

When we compared our model with the GFRα1 structure it was clear that the crucial amino acids for GFL binding are not conserved in GAS1, and it most likely lacks the ability to bind GFL-like ligands, as they all share the same binding mode [21,22]. Indeed Cabrera et al. [8] reported that GAS1 is not able to bind GDNF. While GAS1 lacks the ability to bind GFL-type of ligands, our *in vitro* binding data support the findings by Cabrera et al. [8] that GAS1 can bind RET in a ligand independent manner, and possibly alter the intracellular signalling of RET.

The affinity of GAS1 for RET is significantly lower than that of the GFRα-GDNF ligand complex in solution (*K*_d_ = 12.2 μM *versus* 0.2 nM for GFRα1-GDNF binding RET [31,32]). However on the cell surface the affinity of GAS1 to RET is also likely to be higher as the diffusion is restricted to two-dimensions.

Another possibility is that in some cellular contexts GAS1 would be highly expressed on cell surface, which might boost the binding to RET locally. The exact mechanism of GAS1 on RET signalling remains elusive, but it seems clear that GAS1 has an effect of RET signalling, probably by inhibiting growth-factor dependent signalling [4,8].

Based on the conservation of protein surface features, as mapped on to the GAS1 models, we suggest that the N-terminal domain region defined by α − helices 3–5 might contain a functional binding site (Figure 4), whereas other possible interaction surfaces remain less clear, *e.g.* the very short RGD-peptide motif found in mammalian sequences could be functional, or exist by chance, and so far no biochemical evidence for the function exists.

GAS1 has also been reported to alter SHH-signalling through patched-1 [28,33], indicating that GAS1 has multiple functions. Related to this Pineda-Alvarez et al. [34] and Ribeiro *et al.* [35] reported missense mutations of GAS1 in holoprosencephaly (HPE) patients.In particular Thr200Arg mutation in the second domain of GAS1 Pineda-Alvarez et al. [34] was observed to result in almost complete loss of binding affinity for SHH, hence this domain could also be important for binding to SHH. Also Asn220Lys caused 20% reduction in binding according to Pineda-Alvarez *et al.* [34] and Ala246Ser patient mutations are located in the same domain, while the mutations are some what scattered around the domain and do not cluster together on the surface.

## Conclusions

Our structural data reveal that GAS1 is a flexible two-domain molecule, the flexibility perhaps reflecting its multifunctional properties. The structural arrangement of the domains is clearly different form the compact GFRα structures, suggesting that it has different functional roles. In particular, neither the putative heparan sulphate proteoglycan/RET binding site [22] the known growth factor binding site are conserved in GAS1.

Thus, GAS1 must act on RET in a different way, and together with previous analysis our binding data supports the ligand-independent RET binding by GAS1, while sequence conservation analysis hints at possible sites of functional importance.

## Methods

### Ethics statement

All results of this research were based proteins expressed in cultured *Tricoplusia Ni* or *Spodoptera frugiperda* cells lines. Neither human (human subjects, human material or human data) nor animals (vertebrates or any regulated invertebrates) were used in this experimental research.

### Plasmids, reagents and cell lines

Human GAS cDNA1 in a pCR3.1 plasmid was obtained as kind gift from Prof. Mart Saarma, and the pFastBac (Invitrogen) derivative vector pK509.3 from Prof. Kari Keinänen [36]. Oligonucleotides were purchased from Sigma and Phusion polymerase and PCR reagents were from Finnzymes Inc., *E.coli* DH10Bac-cells, *Tricoplusia Ni* and *Spodoptera Frugiperda* insect cells were from Invitrogen. Serum Free insect cell culture media was purchased from HyClone, gentamycin from Dushefa. Baculovirus production was done according to Bac-to-Bac manual (Invitrogen). SDS-PAGE gels were bought from Bio-Rad. The anti-FLAG monoclonal M1 mouse antibody was from Sigma, the anti-mouse antibody from Santa Cruz biotechnology, 5 ml HisTrap crude Ni-NTA column, size exclusion column Superdex 10/300, and Thrombin protease, 3 M HyBond western-blot membrane, and the ECL reagent were all from GE Healthcare.

### PCR and cloning

Human GAS1 cDNA in pCR3.1 plasmid was used as a template for PCR. The region encoding amino acids 39–317 was amplified, thus omitting the part encoding the native secretion signal at the N-terminus and the predicted GPI-anchor in the C-terminus. The PCR product was subcloned between Not1-Hind III restriction sites to baculovirus pFastBac-derivative vector pK509.3, which has the honey bee mellitin secretion signal and a Flag-tag sequence upstream of the cloning site.

The forward PCR primer was designed to add additional amino acids at the N-terminus for a His_6_-tag and a Thrombin protease cleavage site (LRPHHHHHHLVPRGS).

The PCR primer sequences used for cloning were: 5′ACTTAACTGCGGCCGCATCATCACCATCACCATCTTGTTCCTCGTGGTTCTGCGCACGGCCGCCGCCTCATC-3′ (forward) and 5′-AGATCTTAAGCTTACCTGCGCCCAGGCCCATAG-3′ (reverse). The template was PCR amplified with 5% DMSO to optimize it for a high GC-rich template (here, 81.2 %). PCR cycling conditions were as recommended by manufacturer (Finnzymes Inc). Cleaved and agarose gel purified vector and insert were ligated using T4 ligase (New England Biolabs).

### Virus propagation and Western-blots

The GAS1 construct was transformed to DH10Bac-cells to transpose it as a part of baculovirus shuttle-vector. The resulting DNA was isolated as described in the Bac-to-Bac manual (Invitrogen). Baculoviruses were multiplied by transfecting Sf9 cells on Cellstar (GreinerBio-one) six-well plate at 70 % confluency according to the manufacturer’s instructions (Mirus. USA). In short, 200 μl of serum free HyQ-SFX medium, lacking antibiotics, were placed in microcentrifuge tubes with 6 microliters of TransIt reagent (Mirus), and incubated 20 minutes at room temperature. Two micrograms of bacmid DNA was added to the reactions and incubation was continued for another 20 minutes. Cells were washed with phosphate buffered saline (PBS) pH 7.4, and the medium changed to fresh HyQ-SFX. The transfection mixture was then added to the cells drop-wise. Cells were incubated at +27°C for five hours, after which the medium was changed to serum free SFX medium supplemented with 50 μg/ml Gentamycin. Cells were incubated for five days. The virus production efficacy was estimated by comparing the wells with non-infected control wells; properly infected cells stopped dividing, grew in size, and finally lysed. Virus was passaged typically by infecting 70-90% confluent plates. Passage one was done by adding 2 ml of virus from transfected cells to 70% confluent plate, in a total culture volume of 5 ml. For passages two and three, 90 % confluent plates were made by infecting cells with three to four millilitres of virus from the previous passage in a total volume of 25 ml. Virus propagation was estimated by visual analysis, as described, and by detecting the presence of the flag-tagged GAS1 protein by western blot. Virus propagation was typically continued to at least passage four, in order to get sufficient amount of virus to infect the culture used for protein production.

### Protein production and purification

The GAS1 protein was produced by infecting 200 ml of Tn5 cells, typically at 2 × 10 ^6^ cell density, with 5 ml of high titer virus, typically from passage three or four. 72 h post infection the cells were harvested by centrifugation and the supernatant was collected.

The secreted GAS1 protein was purified from the supernatant by Ni-affinity chromatography. The column was equilibrated with binding buffer containing 20 mM sodium phosphate pH 7.4, 150 mM sodium chloride and 5 mM imidazole, and the protein was eluted with linear 5–500 mM imidazole gradient with the binding buffer. GAS1 protein was detected by SDS-PAGE and identity confirmed by a FLAG-tag Western blot; based on this 1 ml fractions from the peak area were collected (Figure 1).

GAS1 protein containing fractions were detected from the major peak. These pooled and concentrated with a 30 kDa cut-off Amicon spin concentrator (Millipore) for 4000 rpm at +4°C, typically up to 500 μl volume. The buffer was exchanged, to phosphate buffered saline (PBS), pH 7.4, by diluting to 15 ml and repeating the centrifugation step as described.

After buffer exchange, the tags were cleaved off with Thrombin protease at a ratio of ten units per milligram of protein, the cleaved protein was concentrated to a 350 ul final volume, and further purified using size exclusion chromatography with Superdex 10/300 GL column in a buffer containing Tris-buffered saline (TBS) pH 7.4, (25 mM Tris pH 7.4; 150 mM NaCl; 2 mM KCl, pH adjusted with HCl), supplemented with 250 mM NaCl at a 0.5 ml/min flow rate. Fractions of 2 ml were collected and analyzed by SDS-PAGE. The fractions containing correct, approximately 35 kDa protein were pooled and concentrated as previously described.

### Surface plasmon resonance assay

Surface plasmon resonance (Biacore™, GE Healthcare) was used to determine the binding affinity of GAS1 protein to the ectodomain of RET protein (ecRET; R&D Systems, catalog no. 1168-CR-050/CF).

For this purpose ecRET was coupled to a CM5 chip (GE Healthcare) by amide coupling. The chip was ctivated according to manufacturers instructions with 1-ethyl-3-(3-dimethylaminopropyl carbodiimine (EDC)- N-hydroxysuccimide-(NHS) solution (Amine coupling kit, GE Healthcare). The ecRET at 0.25 mg/ml in PBS was diluted 1:10 to 10 mM Na-acetate,pH 5.0 and coupled to the chip at 4000 RU level.

After ecRET was coupled to the chip, the remaining free activated carboxyl groups on the surface were inactivated with 1 M ethanolamine (GE Healthtcare). The buffer used for binding experiment was 10 mM Hepes pH 7.4, 150 mM NaCl (HBS) supplemented with 1 mM CaCl_2_, 0.01% Triton X-100. First flow channel from the chip was used as a blank control channel showing the possible non-specific binding to a non-coated surface. A GAS1 sample concentration series from 2 μM to 32 μM was injected at 20 ul/min for 2 minutes, and after each experiment the chip surface was regenerated with 1 M MgCl_2_ with two 10 μl injections to release the bound GAS1 from RET. The dissociation constant for GAS1 to RET binding was calculated from binding curve calculated fitted from the equilibrium response (R_eq_) values for binding at each concentration (Figure 7).

### Small-angle X-ray scattering studies and multi-angle laser light scattering

The GAS1 protein *ab initio* solution structure was obtained with 0.8 mg/ml protein in 50 mM Tris pH 7.5, 50 mM NaCl buffer. Small-Angle-X-Ray scattering data collection (ESFR, France) was performed with 1 s exposure time per image, and 10 repeats per sample, and these averaged and subtracted from similarly averaged buffer baseline. The measured data was analysed using PRIMUS [37] software and the *ab initio* modelling of the protein was done by DAMMIF/DAMAVER software [38]. The model of the protein with flexible linkers was obtained by using rigid body homology modelling against collected data. Original homology modelling of the domains to GFRα1 was done with Raptor-X server and rigid body modelling of the two-domain structure and modelling of flexible linkers was done with CORAL and BUNCH within the ATSAS software package [39]. Ensemble modelling of SAXS data was done using EOM 2.0 [38,40] via the ATSAS-online server (http://www.embl-hamburg.de/biosaxs/atsas-online/).

### Multi-angle laser light scattering

SEC-MALLS measurements were run at 0.5 ml/min over an S-200 Superdex 10/300 column (GE Healtcare) in 20 mM TRIS pH 7.4, 150 mM NaCl with a Schimadzu HPLC system and MiniDAWN TREOS light scattering detector and Optilab rEX refractive index detector (Wyatt Technology Corporation). Data was analysed with ASTRA 6 software (Wyatt Technology Corporation).

### Circular Dichroism and thermal stability

Circular Dichoism (CD) spectrum at 190–260 nm was collected on a JASCO J-720 instrument. For this experiment the protein was dialyzed against 20 mM Na phosphate pH 7.4, 50 mM NaCl. The GAS1 sample was diluted to 6.5 μM concentration in the same buffer. Measurement was done with a capped 350 μl 1 mm light path quartz cuvette (Hellma-Analytics). Data for thermal denatural analysis was collected at 222 mm wavelength from 20 to 90°C, with one degree steps and 30 second incubation at each temperature.

## Abbreviations

GAS1: Growth arrest specific-1
SAXS: Small-angle X-ray scattering
CD: Circular dichroism
RET: Rearranged during transformation
SEC-MALLS: Size exclusion chromatography and multi-angle light scattering
GFRα: Glial cell-derived neurotrophic factor receptor alpha
GFL: Glial cell-derived neurotrophic factor family ligand
MALDI-TOF: Matrix Assisted Laser Desorption Ionization - Time of Flight
PBS: Phosphate buffer saline
TBS: Tris-buffered saline
SHH: Sonic Hedgehog
GDNF: Glial cell line-derived neurotrophic factor
NCAM: Neural cell adhesion molecule
HBGAM: Heparin-binding growth-associated molecule
